# Ancient Schwannoma of the hard palate. An uncommon case report and review

**DOI:** 10.4317/jced.50950

**Published:** 2013-02-01

**Authors:** Maria L. Gainza-Cirauqui, Asier Eguía-Del Valle, Rafael Martínez-Conde, Juan C. Coca-Meneses, José M. Aguirre-Urizar

**Affiliations:** 1 Oral Medicine Unit. Oral and Maxillofacial Pathology Unit. Faculty of Medicine and Dentistry. UFI 11/25.University of the Basque Country / EHU

## Abstract

Schwannoma or neurilemmoma is an infrequent benign tumor in the oral cavity that originates from the Schwann cells on the neural sheath of the peripheral nerves. Schwannomas are frequently located in the soft tissues of head and neck region, but only a 1 to 12% of them are located in the oral cavity. Some histological variants of schwannoma have been described including the cellular, plexiform, epithelioid, ancient, and melanocytic types. The “ancient schwannoma” is an uncommon variant of this tumor that shows specific histological characteristics, and is rare in the oral cavity with less than 15 cases described on the literature. Most of them were located in the tongue or in the floor of the mouth, being the hard palate an extremely rare localization. We present a new clinical case of an ancient schwannoma with a long time of evolution, arising from the nasopalatine nerve, and located in the hard palate of a 35 year old female. We also review the main clinical and histological characteristics of this pathology.

** Key words:**Ancient schwannoma, neurilemmoma, palate, schwannoma.

## Introduction

Schwannoma or Neurilemmoma is a benign tumor of the neural sheath derived from the Schwann cells (1-3). It was first described in the literature in the year 1910 by Verocay (4), who described the first peripheral nerve tumor. Nevertheless, it was not until 1932 that the term “Schwannoma” was introduced by Masson (5).

Schwannomas most often occur in the fourth and fifth decade of life and seem to have a 1.6:1 female predilec-tion (1-3,6-8). This tumor can appear anywhere in the body, but is more frequently reported in the head and neck and the flexor surfaces of the upper and lower extremities (6-8). Of all the schwannomas, between a 25 and 45% can be found on the head and neck region and only a 1 to 12% of them are located in the oral cavity (6-8). Most of the intraoral cases are found on the upper and lower side of the tongue and vestibule, being rare on the palate (6-8). This tumor most commonly arise in the soft tissues but it can be also found in hard tissues. Intraosseous schwannomas are rare, but when they occur, the mandible is the most common site, particularly in the posterior areas of the body and ramus (6-8).

Clinically, a circumscribed slow-growing, firm and solitary tumor is normally observed (1-3). Most of the cases are asymptomatic and have a long duration and large size because of their lack of symptoms and slow growth (6-9). Pain and paresthesia may be found in some patients, especially in cases of intraosseous localization. Intraosseous schwannoma frequently produces expansion of the affected bone and causes swelling (9,10). Radiographically, it normally appears as a well-circumscribed unilocular radiolucent lesion with thin sclerotic margins (6-10).

The definitive diagnosis is based on the histological and immunohistochemical study (6-10). Several histological variants of schwannomas, have been described. The histological variant of this tumor described as “Ancient schwannoma” shows specific histological characteristics such as degenerative changes and the presence of pleomorphic nuclei, hemorrhagic phenomena or microcyst formation (11,12). Ancient schwannomas tend to be large tumors of long duration and are really rare in the oral cavity. To date, only less than 15 cases of this variant have been reported in this localization (7,11,12).

The treatment of this neoplasm is surgical, with a low recurrence rate (1,2). Malignant transformation has been reported, but is uncommon and many of them are seen in patients with neurofibromatosis type 1 (13). In cases of ancient schwannomas, local recurrence is even more infrequent and malignant transformation has not been described (7,11,12).

After an extended review of the literature, we consider that this could be probably the first clinical case reported of an ancient schwannoma located on the hard palate, or at least the first in which the histology of this histological variant is quite clear in this particular location. The clinical case and the main clinicopathological and therapeutic aspects are described and discussed.

## Case Report

A 35 year old female is referred by her dentist to the Unit of Oral Medicine (Dental Clinic Service of the UPV/EHU) with a slow growing tumor on the anterior portion of the hard palate. Family and medical history was noncontributory and she could not precise the exact time of evolution of the lesion, although, she had no-ticed a marked swelling for at least the past five years. She also pointed out that the lesion probably originated even some years before.

**Figure 1:**
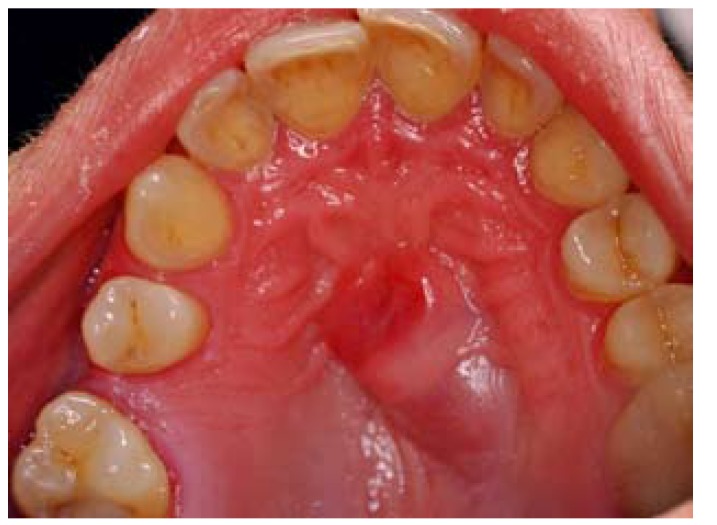
Intraoral view shows an exophytic tumor on the hard palate (mirror image).

**Figure 2:**
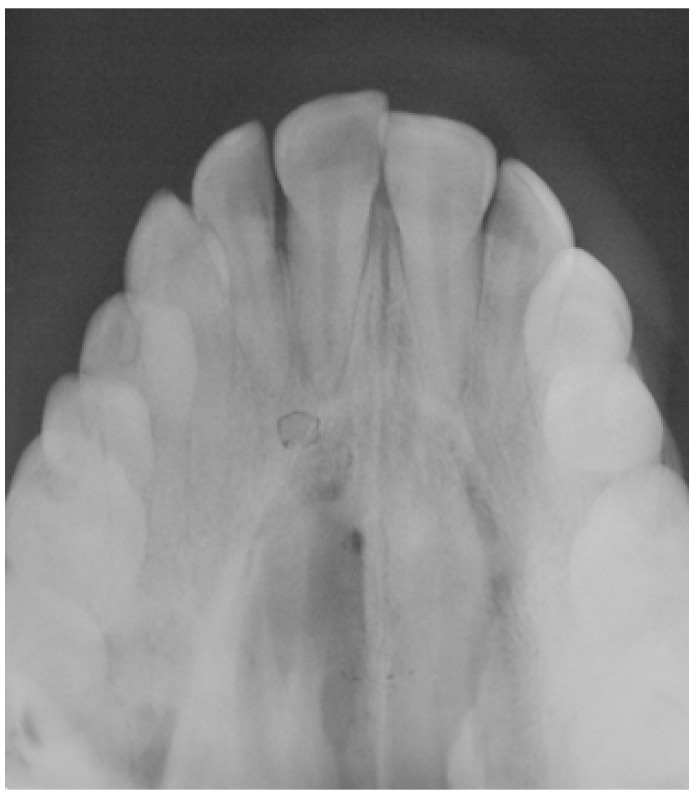
Occlusal radiography showing a not well-defined and slightly more radiolucent area.

On physical examination, a firm, sessile based tumor of the submucosa of 2.0 x 1.5 cm in size was observed in the midline of the hard palate. The surface mucosa was intact except for an erythematous central area (Fig. 1). On the radiographic study, the panoramic radiography did not show any important alteration. Nevertheless, in a maxillar occlusal radiography, a not well-defined and slightly more radiolucent area could be seen in the midline area and the left maxillary palatine process (Fig. 2).

A differential diagnosis was established including salivary gland tumors and different connective tissue tumors and it was decided to perform an excisional biopsy. Previously to the surgical treatment a fine-needle aspiration was performed, but we could not get any tissue sample due to the solid consistency of the lesion. The surgical removal was performed with local anesthesia.

**Figure 3:**
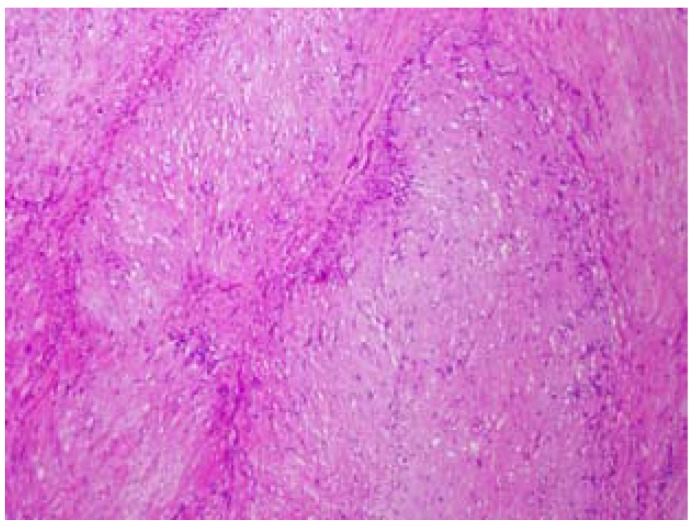
Histopathological characteristics of the tumor. Spindle cell hypercelular areas (Antoni A) and hypocelular and myxoid areas (Antoni B). Degenerative changes can be observed. Hematoxylin and eosin stain 20x.

The tumor was sent for its histopathological analysis. The biopsy showed a well-circumscribed and encapsulated tumor composed by a pleomorphic neoplastic connective proliferation with well-defined areas, and spindle cells with long basophilic nuclei on a peripheral disposition (Antoni A and Verocay bodies) and irregular and hypocellular areas (Antoni B) (Fig. 3). Degenerative changes such as the presence of mild pleomorphism, bizarre nuclei, dilated blood vessels, calcifications and hemorrhagic features were recognized in several areas. The tumor cells were strongly positive to S-100 protein. Based on these findings the diagnosis of ancient schwannoma was established.

The postoperative period was uneventful, and a week after the surgery the sutures were removed. After the resection of the tumor, the patient wore a thermoplastic vacuum formed device to protect the surgical area and to get more comfort during the secondary healing process. After one month a complete healing could be observed and after two year of continuous follow-ups, no recurrence has been observed.

## Discussion

Schwannoma is a benign tumor of unknown cause originated from the Schwann cells of the neural sheath of the peripheral nerves (1-3). Only a 25 to 45% can be found on the head and neck region, with the parapharyngeal space being the most common site (1,2,6-9). A low percentage of these lesions are present in the oral cavity and when this occurs, the tongue is the most frequently involved area (1-3). Most of the intraoral schwannomas are placed in the soft tissues. Intraosseous localization is rare in jaw bones and represent less than 1% of benign primary tumors (1-3).

 Regarding to the ancient schwannoma in the oral cavity, the first report was made by Eversole and Howell in 1971 (14). After that, no more than 15 cases have been reported on the literature (7-12). Most of them were located on the tongue or the floor of the mouth, but it also has been found in other different places such the buccal mucosa, the upper lip or even the submandibular gland (7-12). Our case is almost unique, because of its histopathological features and localization.

Schwannomas most often occur in the fourth and fifth decade of life and seems to have a female predilection (1.6:1) (1,2). Nevertheless, Cohen and Wang (10) performed a revision of the literature including 126 cases of lingual localization and they observed an equal gender predilection (10). Ancient schwannomas occurs in all age groups (7-12), being the younger patient reported 19 years old (7). It is more common between the second and fifth decade of life and more often women, as it was in the case presented in this paper.

Clinically, it normally appears as a slow-growing, solitary exophytic lesion, non-indurated, well circumscribed, with a smooth surface. The size is variable in function to the time of evolution (1-3). Despite the slow growth of this lesion, some giant cases have been reported in the literature (12).

 Most of the cases are asymptomatic and many of them have a long duration and large size because of their lack of symptoms (7-12). The presence of pain, dysphagia or neurological alteration by the compression of the peripheric nerves, can be observed in large sized tumors but also depends on the anatomy of the affected area (7-10). Although the lesion presented in this paper had a long evolution and large size, there were no symptoms. Probably, the absence of symptoms and the long history of the lesion was the cause of the transformation to an “ancient” histological variant of this lesion. The patient could not precise the time of evolution of the lesion, although, she had noticed the swelling for the past five years. This is similar to what occurs in the majority of cases of intraoral ancient variety reported in the literature (7-12).

The differential diagnosis of our lesion included salivary gland tumors and different connective tissue tumors. Among them, slow growing benign and low-grade malignant tumors were first considered. The initial clinical diagnosis was of a salivary gland tumor based on the long-time evolution. Nevertheless, other lesions were not discarded because minor salivary gland tumors are not usually located in the midline. Fine needle aspiratory biopsy can be sometimes of help on the differential diagnosis with other lesions such as cysts, angiomatous lesions or salivary gland tumors (6-10).

Histopathologically, the schwannoma is composed of monomorphic spindle cells with eosinophilic cytoplasm and oval nucleus on hypercellular areas (Antoni A), and myxoid and hypocellular areas (Antoni B). In the Antoni A areas, characteristic eosinophilic formations can be observed, the Verocay bodies (1-3,15). The presence of extensive degenerative changes such as nuclear atypia, hemorrhagic foci or the formation of microcysts and calcification can be the result of a long evolution of the lesions, and suggest the diagnosis of the “ancient” variety of the tumor (1-3,15). Increasing tumor size might result in vascular insufficiency and probably, all schwannomas with a long time of evolution would ultimately show some degenerative changes (1-3,15). In those cases with degenerative changes, an exhaustive but sometimes difficult differential diagnosis must be established with malignant conditions such asleiomyosarcoma, malignant fibrous histiocytoma and others. The study of the whole surgical specimen and the immunohistochemistry analysis is mandatory to avoid misdiagnosis.

The tumor cells of schwannomas are strongly immunoreactive to S-100 protein and are consistently negative for CD117, smooth muscle actin and desmin, and usually negative for CD34 (1-3,15).

Computed tomography (CT) and magnetic resonance are useful tools in evaluating the location and extension of the lesion, as well as the possible relation with adjacent anatomical structures (1-3,15). Due to its location, the panoramic radiography did not provide any relevant information and an occlusal radiography was most helpful. It would have been desirable to have a CT of the case, but it was initially discarded because of the cost of this exploration for the patient.

Complete excision of the lesion, preserving the nerve of origin is the elected treatment (1-3,6-9). No malignant transformation has been described for the Ancient variant of Schwannoma and local recurrence is really low (7-12).

Ancient schwannoma is a slow growing, benign, solitary neoplasm rare in the oral cavity. Histopathological findings are essential for a correct diagnosis. Surgery is the treatment of choice and the rate of recurrence and malignant transformation is exceptional.

## References

[1] Shah AA, Latoo S, Ahmad I, Malik AH, Singh AP, Hassan S (2011). Schwannoma causing resorption of zygomatic arch. J Oral Maxillofac Pathol.

[2] Pfeifle R, Baur DA, Paulino A, Helman J (2001). Schwannoma of the tongue: report of 2 cases. J Oral Maxillofac Surg.

[3] Colreavy MP, Lacy PD, Hughes J, Bouchier-Hayes D, Brennan P, O'Dwyer AJ (2000). Head and neck schwannomas--a 10 year review. J Laryngol Otol.

[4] Verocay J (1910). Zur Kenntnis der Neurofibrome. Beitr Pathol Anat.

[5] Masson P (1932). Experimental and Spontaneous Schwannomas (Peripheral Gliomas): I. Experimental Schwannomas. Am J Pathol.

[6] Buric N, Jovanovic G, Pesic Z, Krasic D, Radovanovic Z, Mihailovic D (2009). Mandible schwannoma (neurilemmoma) presenting as periapical lesion. Dentomaxillofac Radiol.

[7] Subhashraj K, Balanand S, Pajaniammalle S (2009). Ancient schwannoma arising from mental nerve. A case report and review. Med Oral Patol Oral Cir Bucal.

[8] Jahanshahi G, Haghighat A, Azmoodeh F (2011). Intraosseous neurilemmoma of the mandible: report of a rare ancient type. Dent Res J (Isfahan).

[9] López-Carriches C, Baca-Pérez-Bryan R, Montalvo-Montero S (2009). Schwannoma located in the palate: clinical case and literature review. Med Oral Patol Oral Cir Bucal.

[10] Cohen M, Wang MB (2009). Schwannoma of the tongue: two case reports and review of the literature. Eur Arch Otorhinolaryngol.

[11] Humber CC, Copete MA, Hohn FI (2011). Ancient schwannoma of upper lip: case report with distinct histologic features and review of the literature. J Oral Maxillofac Surg.

[12] Zachariades N, Skoura C, Papageorgiou G, Chrissomali E (2001). Giant ancient neurilemmoma of the cervical region: report of case. J Oral Maxillofac Surg.

[13] Shintaku M, Wada K, Wakasa T, Ueda M (2011). Malignant peripheral nerve sheath tumor with fibroblastic differentiation in a patient with neurofibromatosis type 1: imprint cytological findings. Acta Cytol.

[14] Eversole LR, Howell RM (1971). Ancient neurilemmoma of the oral cavity. Oral Surg Oral Med Oral Pathol.

[15] López JI, Ballestin C (1993). Intraoral schwannoma. A clinicopathologic and immunohistochemical study of nine cases. Arch Anat Cytol Pathol.

